# Lifetime prevalence of homelessness in housed people aged 55–79 years in England: its childhood correlates and association with mortality over 10 years of follow-up

**DOI:** 10.1016/j.puhe.2019.12.017

**Published:** 2020-05

**Authors:** P. Demakakos, D. Lewer, S.E. Jackson, A.C. Hayward

**Affiliations:** a Department of Epidemiology and Public Health, Institute of Epidemiology and Health Care, University College London, London, WC1E 6BT, UK; b Collaborative Centre for Inclusion Health, University College London, London, NW1 2DA, UK; c Department of Behavioural Science and Health, Institute of Epidemiology and Health Care, University College London, London, WC1E 6BT, UK

**Keywords:** Adverse childhood experiences, Ageing, Cohort, Homelessness, Longitudinal, Mortality

## Abstract

**Objectives:**

Since 2010, the number of homeless people in the UK has increased, and homelessness in its different types has become a major public health problem. Housed older people with past experience of homelessness are an understudied population that can provide valuable insight into this problem. For this reason, we examined the lifetime prevalence of homelessness and its associations with childhood adversity and mortality in a national sample of older people.

**Study design:**

This is a longitudinal cohort study.

**Methods:**

We studied 6649 housed individuals aged 55–79 years in 2007 from the English Longitudinal Study of Ageing (ELSA). We used logistic regression to model the association between adverse childhood experiences (ACE) and lifetime experience of homelessness (ever been homeless for ≥1 months) and Cox proportional hazards regression to model the prospective association between lifetime experience of homelessness and mortality.

**Results:**

We identified 107 participants with lifetime experience of homelessness. We found a strong graded association between the number of ACE and lifetime experience of homelessness; participants with two ACE had 5.35 (95% confidence interval [CI]: 3.17–9.05) times greater odds of having experienced homelessness than those reporting none. Most ACE were individually associated with lifetime homelessness, but fewer remained so in the mutually adjusted model. Participants with lifetime experience of homelessness had 1.55 (95% CI: 1.01–2.37) times greater risk of mortality over a 10-year follow-up and after adjustment for covariates.

**Conclusions:**

Exposure to childhood adversity is associated with increased risk of experiencing homelessness. Older housed people with past experience of homelessness are at increased risk of mortality.

## Highlights

• Housed older people with past experience of homelessness are an understudied population.
• Childhood adversities are associated with past homelessness in housed older adults.
• This association is strong and follows a dose-response pattern.
• Any previous experience of homelessness raises the risk of mortality in housed older adults.
• Lifetime experience of homelessness appears to be a lifelong risk factor for premature death.

Since 2010, the number of homeless people and rough sleepers in the UK has increased worryingly.^1^, ^2^, ^3^, ^4^, ^5^, ^6^ Homeless people experience extremely poor health and have excessive morbidity and mortality in comparison with the general population.^7^, ^8^, ^9^, ^10^ They are also at greater risk of emergency health care,^11^^,^^12^ whereas geriatric conditions such as functional, sensory and cognitive impairments and frailty are common in the ageing homeless population.^9^^,^^13^^,^^14^ Homelessness is a dynamic phenomenon that takes different forms, such as rough sleeping, living in hostels, sofa surfing and living in temporary accommodation provided by the state,^3^^,^^5^ and follows different patterns over the life course.^15^ The causes of homelessness are complex and include a broad range of structural, societal, and individual factors that interact over the life course.^9^^,^^11^^,^^16^^,^^17^

A fairly large proportion of the population in the Western world has experienced homelessness at least once in their lifetime, with prevalence estimates ranging from 7.7% to 5.1% in the UK and 6.2% to 4.2% in the USA.^18^, ^19^, ^20^, ^21^ Despite these high prevalence estimates and evidence suggesting that temporary homelessness is much more common than chronic homelessness,^22^ people with previous experience of homelessness are underrepresented in health research. Further, limited research has examined homelessness and its life course determinants in general population settings. Previous research has mostly focused on homeless people without comparison with a control group or the general population, and thus cannot directly be informative of differences between homeless and non-homeless people and can only incrementally add to our understanding of the causes and health implications of homelessness at societal level. A better understanding of the pathways to homelessness over the life course is necessary to design more effective policies and prevention strategies and provide more efficient health and social care services to the population.

Adverse childhood experiences (ACE) are common in homeless people^12^^,^^23^ and an established childhood risk factor for homelessness^20^^,^^24^ and many other health and social problems in adulthood.^25^^,^^26^ Studies have examined the association between ACE and lifetime experience of homelessness in adult US samples.^27^^,^^28^ A study has examined this association in a national sample of persons aged 30 years from the 1970 British Cohort Study,^20^ but we are not aware of any such study in the general population of older adults in the UK.

For these reasons, we explored the association between childhood adversity and lifetime experience of homelessness (ever been homeless for 1 ≥ months) in a national sample of housed community-dwelling English adults aged 55–79 years. Our aim was to add to our knowledge of the association between ACE and homelessness over the life course and within the context of general population. Moreover, because the positive association between current homelessness and mortality is strong^7^ and little is known about the effect of past experiences of homelessness on health and survival,^29^^,^^30^ we examined whether housed older people with lifetime experience of homelessness are at increased mortality risk. Our aim was to explore the long-term effect of homelessness on survival at older ages and examine past experience of homelessness as a risk for mortality in safely housed older adults.

## Methods

### Study population

The English Longitudinal Study of Ageing (ELSA) is a population-based observational panel study of community-dwelling older adults and their spouses/partners. The ELSA participants were recruited using stratified random sampling. The baseline ELSA interview (wave 1) took place in 2002-2003 and involved a sample of 12,099 individuals living at private addresses, of whom 11,522 were aged ≥50 years. Follow-up interviews took place at regular intervals every other year. We used data from the one-off ELSA Life History survey that took place in 2007 following ELSA wave 3 (that is the second ELSA follow-up interview). The Life History survey collected retrospective information about the experiences and life circumstances of the ELSA participants including homelessness, from earlier stages of their life before joining ELSA.^31^

Owing to a small number of cases of homelessness among those aged ≥80 years (*n* = 6), we confined our analyses to participants aged 55–79 years. Thus, of the 7855 individuals who participated in the ELSA Life History survey, 6690 were aged 55–79 years and were eligible for inclusion in our study. The analytical sample for the lifetime experience of homelessness analysis included 6649 participants, after the exclusion of 19 participants who did not participate in ELSA wave 3 and 22 with non-valid/missing data on homelessness or childhood adversity. The mortality analysis used a slightly smaller sample of 6366 participants after the additional exclusion of participants who either did not consent to the mortality data linkage (*n* = 136) or had missing data in any of the analysis variables (*n* = 147).

### Assessment of lifetime experience of homelessness

The lifetime experience of homelessness question was part of a list of questions on experiences of living in institutions. Participants were shown a card with eight different non-mutually exclusive options and asked to report if they had ever experienced any of them (‘Can I check, have you ever experienced any of the things on this card?’). The lifetime homelessness option read as follows: ‘…been homeless for 1 month or more?’ Participants who had been homeless for ≥1 months in their life were assigned to the lifetime experience of homelessness category as opposed to everyone else who did not report so.

### Assessment of childhood adversity and covariates

All childhood variables were retrospectively measured and used in our study on an ad hoc basis. Childhood socio-economic position (SEP) was measured using paternal or main carer's occupational class when the participants aged 14 years and the number of books in the household when the participants aged 10 years. We measured the following ten ACE variables: (1) unfavourable childhood circumstances (this included multiple mutually exclusive categories such as having spent most of childhood in single-parent family or living with foster parents or in residential care (children's homes or other institutions), (2) separation from mother for ≥6 months at age ≤16 years, (3) leaving home at young age, (4) severe financial hardship at age ≤16 years, (5) victim of serious physical attack/assault at age ≤16 years, (6) victim of sexual assault at age ≤16 years, (7) physically abusive parents at age <16 years, (8) parents with substance abuse or mental health problems at age <16 years, (9) parents unemployed for >6 months at age <16 years and (10) parents argued or fought very often at age <16 years.

The 10-item ACE list we used taps into the childhood adversity domains that the original ACE study focused: abuse (physical and sexual abuse), household dysfunction (living with parents with substance abuse or mental health problems) and parental separation (separation from mother for ≥6 months).^32^^,^^33^ It also refers to an additional two domains of childhood adversity: economic hardship (prolonged parental unemployment and financial hardship) and experiences with the social care system (any experience of institutional/residential care and foster parents) that have been included in later ACE measures.^34^, ^35^, ^36^, ^37^ The economic hardship domain does not refer to being of low SEP but struggling to make ends meet and having gone through a period of severe financial hardship.^37^ Our ACE inventory also contains items (parents fought very often and having spent most of childhood in a single-mother household) that expand the family relationships/household dysfunction dimension.^36^ Finally, because runaway behaviour^24^ and leaving home early are risk factors for homelessness, our ACE inventory also included an item about leaving home at young age. ACE items similar to ours and inventories of childhood traumatic events have been used in major ageing surveys in the USA, such as the Health and Retirement Study and the Midlife in the US study and have been found to have good validity in older adults.^38^^,^^39^

With the exception of long-term parental unemployment and frequent parental fights, all other ACE variables were strongly associated with lifetime experience of homelessness in the bivariate analysis and were used in the multivariate analysis and the calculation of the ACE summary score. To derive the ACE summary score, we transformed these variables into binary ones (ACE case vs. other). Leaving home at young age was dichotomised around the cut point of ≤18 years (having left home at age ≤18 years vs. other). We also generated two binary childhood circumstances variables (having lived most of childhood with single natural mother vs. not and ever been with foster parents or in residential care in childhood vs. not) that we used instead of the multicategory unfavourable childhood circumstances variable. We generated an ACE summary score by adding together all binary ACE variables. We also measured age, sex, marital status, education (A-level or higher, GCSE/O-level or equivalent and no educational qualifications) and total net non-pension household wealth, which calculation was based on a detailed assessment of wealth including housing wealth and different forms of financial wealth minus any debt owed by the household.^40^

### Mortality

Death registrations spanning the period between the date of the baseline interview in 2007 and April 2018 were obtained from the Office for National Statistics. These data were linked with the interview data for all consenting participants (>97% of the sample).

### Statistical analysis

We first analysed the sample characteristics and ACE in accordance with lifetime experience of homelessness (Table 1, Table 2). We then estimated two logistic regression models of the association between childhood adversity and lifetime experience of homelessness (Table 3, Table 4). The first model examined the potentially cumulative effect of the ACE summary score on the risk of experiencing homelessness at least once in one's lifetime (Table 3). The second model included all variables that were used to derive the ACE summary score (in their original form and before their dichotomisation) and examined their relative importance as predictors of the outcome measure (Table 4). Age and sex were included in both models. Finally, after confirming that the proportionality assumption held (using both plots of the survival curves and the Schoenfeld residuals test), we estimated two Cox proportional hazards regression models of the association between lifetime experience of homelessness and all-cause mortality (Table 5). The first model was adjusted for age and sex and the second in addition for marital status, education and total net household wealth. Time-to-event was calculated in months as the difference between the interview date in 2007 and the month of death or censoring, which was April 2018.

**Table 1:** The sample characteristics by lifetime experience of homelessness (ever been homeless for ≥1 months vs. not) in 6649 ELSA participants.

	Never homeless for ≥1 months	Ever homeless for ≥1 months	*P* value
	N (%)^a^	N (%)^a^	
No. of participants	6542 (98·4)	107 (1·6)	
Mean age, years (SD)	63·3 (8·1)	60·7 (7·4)	≤0.001
Sex			0.71
Men	2938 (44·9)	50 (46·7)	
Women	3604 (55·1)	57 (53·3)	
Married/living with a partner			≤0.001
Yes	4893 (74·8)	52 (48·6)	
No	1649 (25·2)	55 (51·4)	
Education^b^			0.89
A-level or higher including university degree	2781 (42·7)	44 (41·5)	
GCSE/O-level or equivalent qualification	2070 (31·7)	36 (34·0)	
No qualifications	1671 (25·6)	26 (24·5)	
Total net non-pension household wealth^b^			≤0.001
Wealthiest tertile (≥£296,500)	2358 (36·7)	25 (23·8)	
Intermediate tertile (≥£139,850 to < £296,500)	2173 (33·9)	19 (18·1)	
Poorest tertile (<£139,850)	1888 (29·4)	61 (58·1)	
Father/main carer's occupation at age 14 years			0.89
Manager/professional/business owner/administrator	2117 (32·5)	35 (32·7)	
Trade/sales/care services	2039 (31·3)	34 (31·8)	
Plant worker/Casual jobs/Unemployed	2134 (32·5)	32 (29·9)	
Other including retired^c^	252 (3·7)	6 (5·6)	
No. of books in the household at age 10 years			0.24
Enough to fill two or more bookcases (>100 books)	1122 (17·2)	22 (20·6)	
Enough to fill one bookcase (26–100 books)	1933 (29·5)	32 (29·9)	
Enough to fill one shelf (11–25 books)	1571 (24·0)	16 (14·9)	
None or very few (0–10 books)	1630 (24·9)	26 (24·3)	
Other including missing^c^	286 (4·4)	11 (10·3)	

ELSA, English Longitudinal Study of Ageing.

a Unless otherwise stated, this denotes the number of participants in each category (with the respective percent in brackets).

b Education and wealth data were available for 6628 and 6524 participants, respectively.

c The other/missing category was not used in the calculation of the *P value*.

**Table 2:** ACE by lifetime experience of homelessness (ever been homeless for ≥1 months vs. not) in 6649 ELSA participants.

	Never homeless for ≥1 months	Ever homeless for ≥1 months	*P* value
	N (%)^a^	N (%)^a^	
Ever experienced severe financial hardship at age ≤16 years			≤0.001
No/Other incl. missing	5120 (78·3)	59 (55·1)	
Yes	133 (2·0)	4 (3·7)	
Yes, but respondent did not report the age that this happened	213 (3·3)	14 (13·1)	
Missing^b^	1076 (16·4)	30 (28·1)	
Parents unemployed for >6 months when participant aged <16 years			0.70
No	5086 (77·7)	71 (66·4)	
Yes	364 (5·6)	6 (5·6)	
Missing or did not complete the childhood experiences questionnaire^b^	1092 (16·7)	30 (28·0)	
Childhood life circumstances^c^			≤0.001
Lived most of childhood with both natural parents	5617 (85·9)	75 (70·1)	
Lived most of childhood with natural mother and stepfather	93 (1·4)	1 (0·9)	
Lived most of childhood with natural father and stepmother	25 (0·4)	1 (0·9)	
Lived most of childhood with single natural mother	356 (5·4)	10 (9·4)	
Lived most of childhood with single natural father	62 (1·0)	1 (0·9)	
Lived most of childhood with grandparents or other	197 (3·0)	3 (2·8)	
Ever lived in residential care or with foster parents in childhood	192 (2·9)	16 (15·0)	
Age stopped living with parents/guardians to live on one's own or establish one's own home			≤0.001
>20 years	3961 (60·5)	41 (38·3)	
19–20 years	1258 (19·2)	25 (23·4)	
18-17 years	947 (14·5)	25 (23·4)	
15–16 years	240 (3·7)	10 (9·3)	
<15 years	46 (0·7)	4 (3·7)	
Missing^b^	90 (1·4)	2 (1·9)	
Separated from mother for ≥6 months at age ≤16 years			≤0.001
No	5608 (85·7)	71 (66·4)	
Yes	934 (14·3)	36 (33·6)	
Ever been a victim of serious physical attack/assault at age ≤16 years			≤0.001
No	5364 (82·0)	69 (64·5)	
Yes, at age ≤16 years	82 (1·3)	7 (6·5)	
Yes, but respondent did not report the age that this happened	42 (0·6)	3 (2·8)	
Missing or did not complete the childhood experiences questionnaire^b^	1054 (16·1)	28 (26·2)	
Ever been a victim of sexual assault at age ≤16 years			≤0.001
No	5236 (80·0)	68 (63·6)	
Yes, at age ≤16 years	221 (3·4)	7 (6·5)	
Yes, but respondent did not report the age that this happened	33 (0·5)	4 (3·7)	
Missing or did not complete the childhood experiences questionnaire^b^	1052 (16·1)	28 (26·2)	
Parents had substance abuse or mental health problem(s) when participant aged <16 years			≤0.001
No	5145 (78·7)	65 (60·7)	
Yes	336 (5·1)	12 (11·2)	
Missing or did not complete the childhood experiences questionnaire^b^	1061 (16·2)	30 (28·1)	
Parents physically abused the participant at age <16 years			≤0.001
No	5293 (80·9)	69 (65·5)	
Yes	196 (3·0)	10 (9·3)	
Missing or did not complete the childhood experiences questionnaire^b^	1053 (16·1)	28 (26·2)	
Parents argued or fight very often when participant aged<16 years			0.036
No	4314 (65·9)	53 (49·5)	
Yes	1111 (17·0)	23 (21·5)	
Missing or did not complete the childhood experiences questionnaire^b^	1117 (17·1)	31 (29·0)	
No. of adverse childhood experiences at age (range:0–9)			≤0.001
0	3834 (58·6)	34 (31·8)	
1	1783 (27·2)	28 (26·2)	
2	623 (9·5)	28 (26·2)	
3	224 (3·4)	12 (11·2)	
≥4	78 (1·1)	5 (4·6)	

ELSA, English Longitudinal Study of Ageing; ACE, adverse childhood experiences.

a Unless otherwise stated, this denotes the number of participants in each category (with the respective percent in brackets).

b The missing category was not used in the calculation of the *P value*.

c The childhood life circumstances categories were exclusive; participants can be in only one of these categories.

**Table 3:** The association between the ACE summary score and lifetime experience of homelessness (ever been homeless for ≥1 months vs. not) in 6649 ELSA participants aged 50–79 years.

	Odds ratio (95% CI)
Age	0·96 (0·94–0·99)
Sex	
Men	1·00 (reference)
Women	0·85 (0·57–1·25)
No. of adverse childhood experiences	
0	1·00 (reference)
1	2·05 (1·26 to 3·34)
2	5·35 (3·17 to 9·05)
3	6·86 (3·33 to 14·14)
≥4	11·24 (4·51 to 28·01)

ELSA, English Longitudinal Study of Ageing; ACE, adverse childhood experiences; CI, confidence interval.

**Table 4:** The association between individual ACE variables and lifetime experience of homelessness (ever been homeless for ≥1 months vs. not) in 6649 ELSA participants aged 50–79 years.

	Odds ratio (95% CI)^a^
Spent most of childhood with a single natural mother	
No	1·00 (reference)
Yes	1·91 (0·97 to 3·78)
Ever lived with foster family or in residential care (children's home or other institutions)	
No	1·00 (reference)
Yes	2·37 (1·20 to 4·67)
Age stopped to live with parents/guardians to live on one's own or establish one's own home^b^	
>20 years	1·00 (reference)
20 to 19 years	1·51 (0·89 to 2·55)
18 to 17 years	2·06 (1·22 to 3·48)
15–16 years	2·11 (0·99 to 4·49)
<15 years	N/A^c^
Separated from mother for ≥6 months at age ≤16 years	
No	1·00 (reference)
Yes	2·07 (1·26 to 3·38)
Ever been a victim of serious physical attack/assault at age ≤16 years^b^	
No	1·00 (reference)
Yes, at age ≤16 years	3·14 (1·22 to 8·10)
Yes, but respondent did not report the age that this happened	N/A^c^
Ever been a victim of sexual assault at age ≤16 years^b^	
No	1·00 (reference)
Yes, at age ≤16 years	1·16 (0·47 to 2·88)
Yes, but respondent did not report the age that this happened	N/A^c^
Parents had substance abuse or mental health problem(s) when participant aged <16 years^b^	
No	1·00 (reference)
Yes	1·31 (0·65 to 2·65)
Parents physically abused the participant at age <16 years^b^	
No	1·00 (reference)
Yes	1·21 (0·53 to 2·79)
Ever experienced financial hardship at age ≤16 years^b^	
No	1·00 (reference)
Yes	N/A^c^
Yes, but respondent did not report the age that this happened	4·37 (2·31 to 8·29)

ELSA, English Longitudinal Study of Ageing; ACE, adverse childhood experiences; CI, confidence interval.

a The odds ratios presented here are adjusted for age and sex and mutually adjusted for all ACE variables included in this table.

b For clarity purposes, the odds ratios for categories representing missing values and non-valid responses are not shown.

c Category too small (<5 participants) to confidently calculate the odds ratio.

**Table 5:** The association between lifetime experience of homelessness (ever been homeless for ≥1 months vs. not) and mortality in 6366 ELSA participants aged 50 to 79 years.

	Never homeless for ≥1 months	Ever homeless for ≥1 months
No. of participants	6262	104
No. of cases	1064	22
Mean follow-up time in years (median)	10·1 (10·8)	9·8 (10·8)
Person-years	63,071	1014
Incidence per 1000 person-years (95% CI)	16·9 (10·9 to 13·2)	21·7 (14·3 to 33·0)
Cox proportional hazards analysis		
Model 1^a^	1·00 (reference)	1·90 (1·24 to 2·90)^c^
Model 2^b^	1·00 (reference)	1·55 (1·01 to 2·37)^c^

ELSA, English Longitudinal Study of Ageing; CI, confidence interval.

a Model 1 was adjusted for age and sex.

b Model 2 was adjusted for age, sex, education and total net non-pension household wealth.

c The estimates are hazard ratios (95% CI).

## Results

**Fig. 1:**
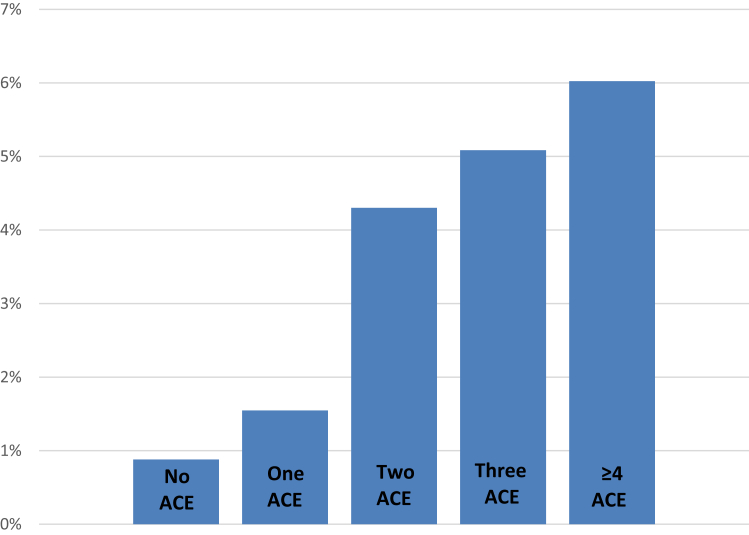
Lifetime prevalence of homelessness by the number of adverse childhood experiences (ACE) in 6649 ELSA participants aged 55–79 years. ELSA, English Longitudinal Study of Ageing.

We identified 107 participants who had been homeless for ≥1 months at some point in their life. Compared with those who had never been homeless, participants who had experienced homelessness were less likely to be older, married and wealthier (Table 1). They were also more likely to having been through the social care system in childhood, spent most of their childhood in a single-mother family, stopped living with their parents/guardians at a younger age, been separated from their mother for >6 months or experienced financial hardship or physical or sexual attack/abuse at age ≤16 years, and had abusive parents with mental health and substance abuse problems at age <16 years (Table 2). The association between the ACE summary score and lifetime prevalence of homelessness was graded (Fig. 1). The multivariable logistic regression analysis (Table 3) confirmed that the likelihood of lifetime homelessness increased along with the count of ACE after adjustment for age and sex. The risk of lifetime experience of homelessness was increased even among those who reported only one ACE, odds ratio (OR): 2.05, 95% confidence interval (CI): (1.26–3.34). Regarding specific ACE, the regression analysis indicated that after adjustment for age and sex and mutual adjustment for all ACE items, lifetime experience of homelessness remained significantly associated with unfavourable living arrangements, long-term separation from mother and experience of physical attack (Table 4).

Regarding mortality, we observed 1086 deaths over a mean follow-up time of 10.1 years among 6366 of our participants. Twenty-two of these deaths were observed among the 107 participants who had experienced homelessness at least once in their lifetime. The Cox regression models indicated that participants who had experienced homelessness at some point in their life had 90% increased risk of dying compared with those who had never been homeless after adjustment for age and sex (Table 5). Additional adjustment for education and total net household wealth partially explained the association and decreased the hazard ratio to 1.55 (95% CI: 1.01–2.37).

## Discussion

In a national sample of people aged 55–79 years, we found that ACE were strongly associated with the risk of experiencing homelessness at some point in one's life. On the basis of the Bradford Hill criteria for causation,^41^ the graded ‘dose-response’ pattern and magnitude of the association between the ACE summary score and lifetime experience of homelessness may support a causal association. We also found that older adults who have ever been homeless were at increased mortality risk.

### Strengths and weaknesses

The use of a national sample of older housed community dwellers, the measurement of many different ACE and SEP markers, the long 10-year follow-up and its novelty are strengths of our study. Nevertheless, our study also has limitations that need to be acknowledged. First, we could not establish when our participants became homeless or the type of homelessness that they had experienced. But given that a main exposure, ACE, refers to childhood, and a main outcome is mortality at older ages, there is still a strong temporal sequence in the analyses. Second, childhood measures and homelessness information have been collected in retrospect and may be susceptible to recall bias. Childhood SEP measures such as paternal occupational class at 14 years of age have successfully been used in previous studies to predict morbidity and mortality.^42^^,^^43^ Nevertheless, the retrospective measurement of childhood experiences of abuse poses a particular problem as it is known to be problematic with many false negatives.^44^ Third, we lacked data on dimensions of childhood adversity that were standard part of the original ACE scale, such as emotional/psychological abuse and incarceration experiences in the household. Fourth, by design, the baseline ELSA sample included older people who were living in private addresses and this reduced the applicability of our findings to the currently homeless population. Fifth, our study included a relatively small number of cases of lifetime homelessness and this resulted in uncertainty about the effect sizes.

### Interpretation of findings

Our findings on the association between childhood adversity and homelessness concur with those of two reviews.^12^^,^^23^ One of these reviews focused on the prevalence of physical and sexual abuse in childhood among young homeless people in the USA and Canada. They found much higher rates of physical and sexual abuse in homeless men and women compared with the general population.^23^ The other review examined risk factors for homelessness in US veterans and concluded that ACE are moderately associated with the risk of homelessness.^12^ Together with these studies, our findings suggest that ACE is associated with homelessness in different settings and across generations.

Our study adds to the literature in different ways. Next to evidence suggesting that adversities tend to cluster together and are interrelated, our findings indicate that experiences of multiple severe adversities in childhood likely put people at considerably increased risk of homelessness. But we also found that having only one ACE was sufficient to elevate one's risk of subsequent homelessness. Further, our findings delineate the existence of several childhood adversity pathways leading to homelessness. One of these pathways is related to living circumstances in childhood and refers to lacking a two-natural-parent family, limited family resources and decreased provision to the child. Having left home at age ≤18 years is an important dimension of this pathway and likely a risk factor for subsequent experience of homelessness. A second pathway is related to abuse, traumatic events and highly stressful experiences. We can speculate that inadequacy of socioemotional resources to deal with trauma and mental health problems stemming from it possibly are parts of this pathway.

Commonly used childhood SEP measures such as paternal occupational class at age 14 years, long-term parental unemployment and number of books in the household at age 10 years were not associated with lifetime homelessness. This may be because these variables might not capture the levels of extreme disadvantage that are predictive of homelessness. Furthermore, these measures were not directly relevant to participants who did not spend most of their childhood with both natural parents. We also found that the risk of lifetime homelessness was not different in men and women in our data, and this is discrepant with statistics suggesting that three-quarters or more of rough sleepers are male.^45^ Notwithstanding the possibility of significant generational differences in experiences of homelessness and cohort effects, we can speculate that this discrepancy might be related to our focus on lifetime experience of homelessness, which is broader than current homelessness and rough sleeping, and includes ‘hidden’ forms of homelessness and episodes of transient homelessness. Furthermore, cases of homelessness in our study are by default survivors who managed to overcome homelessness. It is possible that survivorship in this context might be affected by factors that favoured women over men, such as men's greater exposure to long-term and more severe homelessness. Relevant to this speculation is our finding of an inverse association between age and the risk of lifetime homelessness, which is suggestive of the powerful impact of homelessness on survival and the decreased chances people with lifetime experience of homelessness have to reach old age.

To our knowledge, our study provides one of the first estimates of mortality risk in people who have ‘recovered’ from homelessness in the UK. Our findings suggest that having gone through the experience of homelessness is an important risk factor for mortality even among resilient older people who managed to overcome homelessness and are currently in stable housing. We found that people who had experienced homelessness had almost double the risk of all-cause mortality after adjustment for age and sex. Previous studies of older individuals who were homeless or living in shelters and hostels reported comparable mortality estimates.^46^, ^47^, ^48^ Nevertheless, we anticipate that our findings likely are a conservative account of the true association between having experienced homelessness and mortality over the life course. This is because by design, our study ignored the impact of previous experience of homelessness on mortality risk at age <55 years, where many of the homelessness-related deaths occur.

### Conclusion

Severe adversity in childhood is associated with lifetime experience of homelessness in our sample of older housed community dwellers. Our findings add to the literature and can be used to inform strategies and initiatives to prevent homelessness and help vulnerable individuals and disadvantaged communities. They also suggest that people who have ‘recovered’ from homelessness remain at increased mortality risk, even after accounting from material deprivation in adulthood. This is a finding with major implications for practice as it delineates a ‘hidden’ population at risk and adds to the argument for the need to have adversity- and trauma-informed practice. There is need for continued support across the life course for people who have been homeless even after they become securely housed. Our work emphasises the importance of the life course dimension of homelessness for population health and pushes the boundaries of the current conceptualisation of homelessness, from a problem of a minority of marginalised people to that of a lifetime risk factor for mortality in the general population. Future research should explore the pathways through which ACE lead to an increased lifetime risk of homelessness, build better life course models of homelessness and add to the exploration of the impact of lifetime homelessness and hidden forms of homelessness on morbidity and mortality.

## Author statements

### Funding

The English Longitudinal Study of Ageing is supported by the 10.13039/100000049National Institute on Aging (Grants 2R01AG7644 and 2R01AG017644-01A1) and a consortium of the UK government departments coordinated by the 10.13039/501100000269Economic and Social Research Council. The National Institute on Aging and the consortium of the UK government departments had no role in the design and conduct of this study; collection, management, analysis, and interpretation of the data; and preparation, review, or approval of the manuscript. Professor Hayward is a 10.13039/501100000272National Institute for Health Research (NIHR) Senior Investigator and codirector of the UK Prevention Research Partnership (UKPRP) – ActEarly: a City Collaboratory approach to early promotion of good health and wellbeing (RC grant reference MR/S037527/) that is administered by MRC as part of a multifunder alliance including the 10.13039/100010269Wellcome Trust. The views expressed in this article are those of the authors and not necessarily those of the NIHR, the Department of Health and Social Care, or the funders of the UKPRP.

### Ethical approval

The English Longitudinal Study of Ageing was approved by the National Research Ethics Service (London Multicentre Research Ethics Committee (MREC/01/2/91)).

### Competing interests

None declared.

### Patient consent

Informed consent was obtained from all participants.

### Data sharing

The raw data are available from the UK Data Service.
